# Is Electricity as a Therapeutic a Humbug?

**Published:** 1876-04

**Authors:** James B. Baird

**Affiliations:** Lecturer on Diseases of the Nervous System in the Atlanta Medical College. Atlanta, Ga.


ATLANTA

MEDICAL AND SURGICAL JOURNAL

VOL. XIV.] APRIL—1876. [No. 1

### Original Communications.

### IS ELECTRICITY, AS A THERAPEUTIC AGENT, A HUMBUG?

BY JAMES B. BAIRD, M.D.,
Lecturer on Diseases of the Nervous System in the Atlanta Medical College.

*Read before the Atlanta Academy of Medicine.*

The answer to this question, to those gentlemen who have tested the virtues of electricity, possesses but little interest, for they have their own observations to support its claims and to testify in its behalf; but with the hope of arresting the attention of that unfortunately large class of physicians, who have through prejudice, or from other causes, failed to avail themselves of so valuable a means for the relief of suffering and the cure of disease, I propose to record my experience with the use of the remedy.

It is not my purpose to enter into an extended history of electro-therapeutics; that would be interesting in its way, but the object of this paper is simply to present, as succinctly as possible, a few bald facts, and to leave the members of the Academy to draw their own inferences.

The histories of electricity and of electro-therapeutics are inseparable. Every advance step in the one has been marked by progress in the other, from its earliest introduction as a remedial agent; but the period of most interest to the practical practitioner of medicine dates from the discoveries of Galvani and Faraday. The scientifically educated physician has ever been slow to recognize the therapeutic value of electricity; yet as the knowledge of its powers has increased, and the modes of its production and application have been improved, its popularity has steadily extended, and I confidently anticipate the day, not far distant, when electricity, numbering as it does among its supporters many of the ablest minds in the profession, not only in this country, but in Europe, shall be accorded the high place it so richly merits, and shall be accepted by the mass of intelligent physicians as indispensable in the treatment of many diseases.

The scepticism evinced by the majority of physicians in respect to the remedy has left it, to a large extent, in the hands of unprincipled charlatans and ignorant empirics, who have at all ages abounded, and who have done much to bring it into undeserved disrepute among the educated and intelligent. The mysterious power of so subtile a force furnishes ample ground for appeals to the imaginative faculties and the superstitions of the weak, and the often surprising results that have followed even its indiscriminate use, lend a color of truth to their reckless assertions.

Dr. Moritz Meyer (*Meyer's Medical Electricity*) says: "All those who have had an opportunity of observing closely the brilliant services of Remak, must indorse the words of Graefe, that 'Remak, by introducing the constant current into the practice of medicine, enriched it with an invaluable treasure, whose aid in numerous otherwise incurable cases is incalculable.'" Professor William A. Hammond, M.D., (*Journal of Psychological Medicine*, July, 1872,) quotes Dr. Francis E. Anstie, of London, as saying: "I am now fully able to speak with far greater assurance of the positive value of electricity as a remedy for neuralgic pain. I shall make bold to say that nothing but the general ignorance of the facts can account for the extraordinary supineness of the mass of English practitioners with regard to this question." Dr. Hammond adds: "This is true of America. Many of our physicians know almost nothing of the great benefits to be gained by this agent, and have a groundless scepticism of all that is said in its favor."

The inquiry is frequently made, In what diseases is electricity indicated? It is difficult to answer this question by giving a classified list of the diseases in which it has been found beneficial, but, as a contribution in this direction, I append a few extracts from the writings of recognized and well-known authorities, and a few cases taken from my note-book for the past year.

Prof. John B. Biddle (*Biddle's Materia Medica*), referring to the action of electricity, says: "In the form of electro-magnetism it is a powerful excitant in the coma resulting from narcotic poisons, and in asphyxia generally, and is probably the most active remedy that can be exhibited in these cases." Prof. Niemeyer (*Text Book of Practical Medicine*) affirms that "in the constant current we have a means more powerful than any other of modifying the nutritive condition of parts that are deeply situated."

Dr. Hammond (*Diseases of the Nervous System*) speaking of the treatment of neuralgia, uses these words: "But above all means, not only for relieving the pain of any particular paroxysm, but also for effecting a permanent cure, electricity stands first. I have cured a number of severe cases of nearly every kind of neuralgia by the aid of electricity when other means had entirely failed." This same author says of the treatment of cerebral congestion: "The constant galvanic current posesses the power of contracting the blood vessels when so used as to stimulate the sympathetic nerve. This property of the constant current was first pointed out by Barnard, Waller and Budge; but its demonstration by the ophthalmoscope was first made by myself. Observation with this instrument, while the current is acting, shows that the vessels of the retina contract, and hence there can be no doubt that this action is produced upon those of the brain. A similar effect is produced by passing the current directly through the brain, the poles being applied to the mastoid processes. The good effects of this practice are well marked, a few applications being often sufficient to abolish the vertigo and unpleasant feelings in the head, and to restore mental and physical activity." In reference to the treatment of paralysis, he uses the following language: "But no agent is so valuable in hemiplegia as electricity, and amendment almost invariably follows its use, even in old cases, in which there are tonic contractions. As regards the restoration of sensibility, it will generally be found to be less difficult than the removal of the motor paralysis. The treatment consists mainly in the use of the electric wire-brush. I have succeeded in curing almost complete anæsthesia, from cerebral hemorrhage, by this treatment alone. In recent cases it will almost invariably prove effectual. Hyperæsthesia may be similarly managed. * * * In hysterical anæsthesia electricity is almost a specific. I have never seen a case resist it." In an article on spinal irritation, the same writer affirms: "There is a remedy which apparently either contracts or enlarges the blood vessels (according to its mode of application), and which is more efficacious in removing spinal irritation than any other with which I am acquainted, and that is the constant galvanic current."

Allan McLane Hamilton, M.D., (*Clinical Electro-Therapeutics*) says: "I indorse electricity as a very valuable remedy in certain diseases. * * * As a therapeutic means in nearly all forms of nervous disease, electricity is invaluable."

Prof. Austin Flint, M.D., (*Flint's Practice of Medicine*) treating of the management of paralysis, uses these words: "The judicious employment of electricity as a therapeutic agent in cases of paralysis is undoubtedly of great importance. * * * The ends to be attained by electricity, it should be kept in mind, are the restoration of the ability of the paralyzed muscles to respond to the will, and the maintenance of the circulation, together with healthy nutrition within the affected organs. * * * In cases in which the paralyzed muscles fail to respond to the electrical current from defective innervation, as an immediate or speedy result of morbid conditions affecting the spinal cord or nerves, recovery is not to be expected so long as the paralysis is sustained by the persistence of these morbid conditions. Yet, under these circumstances, the judicious employment of electricity may promote circulation and nutrition, and thus tend to keep the muscles in a state of preparation for resuming their capacity to act in obedience to the will, when the conditions sustaining the paralysis are diminished or removed. A response to the electrical current, as regards contractility or sensibility, or both, is evidence of a returning innervation, and then the persevering employment of electricity may prove of great service. * * * Much benefit, in cases of poisoning from lead, may be hoped for from electricity, and its employment may do much toward hastening recovery in cases of functional paralysis. The foregoing remarks have had reference chiefly to the paralysis of motion. The judicious application of electricity in cases of paralysis of sensation is often useful. The success of electrical treatment in paralytic affections will depend on the exercise of judgment in determining the indications for it, and of tact in its employment. The advantage of experience in the practical application, together with the time which it requires, renders it desirable that some members of the profession should give it special attention. Unfortunately the employment of electro-therapeutics in this country is, for the most part, in the hands of uneducated practitioners, who know but little of the agent which they employ, and still less of the human organism." Referring to the treatment of neuralgia, Dr. Flint says: "Electricity has been found often useful and sometimes efficacious. I have met with a case of intense paroxysms of pain in the course of the sciatic, arising from intra-pelvic malignant disease, in which, for a time, more relief was afforded by the electro-magnetic current than by all other palliative measures. Electricity, applied in conjunction with acupuncture, has been successfully resorted to in some rebellious cases. The palliative effect of electricity is probably due to a temporary blunting of the excitability of the nerve or nerves to which the pain is referred. Their capacity either to originate or to conduct painful sensations is diminished or annulled by passing through them the electric current."

I have quoted the above author at length, on account of his well-known conservatism, and as a representative of the most advanced views relative to the general practice of medicine in this country. I think, however, that he does not tell the whole story in his attempt to explain the *modus operandi* of electricity in relieving neuralgia. Its simply benumbing effect upon sensative parts is not the only influence it exerts in overcoming hyperæsthesia.

Dr. M. Meyer, Berlin, (*Meyer's Medical Electricity*, translated by Hammond,) speaking of the treatment of the various paralyses, says: "The electric current has, from the oldest times, been extensively employed; indeed, it is, on account of its inlying qualities, to be used in preference to all other means." He arrives at the following conclusions respecting the properties of electricity in this connection: It is a stimulus. It increases the supply of blood in the irritated part of the body. It augments the temperature, and increases the volume of the part to which it is applied. It enhances the contractile energy of the vascular walls. It counteracts the secondary changes occurring in inactive nerves and muscles. It is capable of restoring to nerves and muscles their lost functional power. It is capable of developing a supplementary function in muscular fibres not yet paralyzed. It acts directly on the brain and spinal marrow. This same acknowledged authority on this subject places electricity "far above all other means for the treatment of peripheral neuralgia."

The literature of the subject is replete with such testimony as has been adduced, and volume upon volume might be presented, but it is unnecessary to offer further evidence of this character. It has been shown that men who stand second to none in their intellectual and scientific attainments, give to this great remedy, which they have thoroughly and conscientiously tested, their unqualified indorsement, and it only remains for me to report briefly a few cases illustrative of its practical application in my own hands:

Mrs. D., æt. about fifty years, cancer of the breast, too extensive to admit of an operation for its removal. Breast hypertrophied, indurated, extensively ulcerated, and livid. Pain constant and intense, darting through the body; intercostal neuralgia and subscapular pain.

She had been in the habit of taking large doses of morphine every two or three hours for several months. This alleviated but did not remove the pain, and secured to her usually a brief, uneasy sleep. The morphine was totally suspended. I applied a mild galvanic current through the breast for ten minutes; also to the affected nerve trunks, with entire relief of pain, followed by a sound, undisturbed night's sleep, a luxury unknown for months. A slight return of the pain next morning was promptly checked by a repetition of the current. She remained in the city for a week, receiving two applications daily. She continued free from all pain and slept well. No opiate was taken. At the end of this time, she returned to her home—a hundred miles distant—but came back to this city after ten days, with recurrence of pain. Three or four applications of the current during the week, again brought her entire immunity from suffering. The congestion of the vessels was lessened, the lividity of the integument gradually receding from the edges. She was advised to procure a galvanic battery, and use it for the remainder of life, or, as long as it should be required. Since that time I have not heard from her.

Clara H., colored, æt. thirty-five years—spinal irritation of five years standing. (I shall not stop here to discuss the pathology of spinal irritation, so-called, or to defend the use of the term; it represents a condition, the symptoms of which are sufficiently well understood by the profession.) This patient suffered from intercostal neuralgia; intense paroxysms of pain in the region of the right ovary, shooting down the thigh, and various visceral derangements. She had been frequently treated for "ovarian irritation," but continued to grow worse. Careful examination revealed the existence of a tender spot on the spine in the dorsal region. She then remembered that she had been, for a long time, unable to lean back in her chair, on account of the pain that the pressure occasioned. The treatment consisted in the use of the constant galvanic current to the spine, and to the seats of pain elsewhere. Strychnia gradually increased to the point of tolerance, and two small blisters successively applied to the tender vertebræ. The pain was immediately relieved by the galvanization, and subsequently, upon any intimation of its return, was dissipated by passing the current. The tenderness gradually subsided. This treatment was continued for a few weeks, when she was dismissed, cured.

Miss Carrie G., æt. nineteen years—spinal irritation of a few weeks duration. Extreme paroxysms of pain in both iliac regions, along certain intercostal nerves, and in both arms, with irregular respiration, and contraction of the muscles of the arms. A blister had already been applied over the tender vertebræ. The constant galvanic current was used to the spine, and the constant and interrupted galvanic currents to the painful regions. The pain was promptly and invariably relieved by each application; the contracted muscles were completely relaxed, and the respiration became regular. This treatment was persevered in once or twice daily, as demanded, for two weeks, when the patient was dismissed, cured.

Mr. J. L. H., æt. forty-five years—supra-orbital neuralgia. Had been treated by various means without benefit. The constant current passed through the affected nerves secured instant relief. This treatment was resorted to twice daily for five days. No return of the pain followed its discontinuance.

Mary D., colored, æt. thirty years—facial neuralgia, from sleeping in a damp basement. A single application of the constant galvanic current to the seat of the pain relieved.

Miss Y., æt. eighteen years—intercostal neuralgia. She was entirely relieved by a single application of the constant galvanic current to the affected nerves.

Mr. M. S., æt. twenty-eight years—cervico-occipital neuralgia, violent. Has been subject to similar attacks; sometimes in bed from them for several days. A mild galvanic current afforded instantaneous relief. No return.

Mr. W. N. J., æt. thirty-three years—lumbago. Pain very great; inability to bend the body; walked with great difficulty, his body being supported by his hands resting upon his hips; is subject to such attacks. Opium had been freely taken, without relief. Galvanization of the affected region immediately and permanently dissipated all of the symptoms.

Mr. M., æt. thirty years—syphilitic tumor of the brain, accompanied by excruciating circumscribed pain in the head, total loss of vision, partial paralysis of the left side, and constant tremor of the left arm, with inability to sleep, and frequent epileptiform convulsions, limited to one side of the body, daily. The persistent insomnia, from pain, was a source of great discomfort and disadvantage to him. He was already on active anti-syphilitic treatment. In addition I used the constant galvanic current to the brain and to the left arm. The result was, without exception, relief of pain, followed by refreshing sleep, and after a few applications the tremor in the arm ceased. He passed from under my observation in a few weeks, and I do not know his subsequent history.

(I have since been reliably informed that improvement in this case has been steadily progressive. Anti-syphilitic treatment has been vigorously pursued, and complete recovery, except so far as relates to vision, is probable.)

Mr. J. H. M., æt. fifty years—functional paralysis of the elevator muscles of the feet, accompanied by circumscribed anæsthesia of each leg. (The term functional paralysis possesses a practical signification that warrants its use in describing this case. I do not believe it is a purely functional affection, if, indeed, there are any such—but a discussion of its pathology would be out of place here.) Treatment, the constant galvanic current to the lower part of the spine and the faradic current to the affected muscles; the same applied by means of a wire brush to the anæsthetic spots, and small doses of strychnia and phosphide of zinc. The improvement after each application was marked. After ten applications he was dismissed with motor and sensory power completely restored.

The following case, the last that I shall present, is, so far as I know, unique: Geo. D., æt. three months, was brought to my office by his mother, in April last, with the statement that there was some trouble about his throat, which interfered with his breathing. The difficulty commenced six weeks before, without obvious cause, and had gradually increased. She stated that he had nearly choked to death on several occasions during sleep, and consequently she had been compelled to keep him constantly in her arms for several days and nights. During each act of inspiration a loud stertorous sound was emitted, which his mother called snorting. When placed on his back inspiration was impossible, and his countenance was expressive of great terror. Breathing was freer when he lay on his face. There was no history or evidence of cerebral derangement, or of the reception of any injury. The child was remarkably bright and intelligent for his age. The uvula was slightly congested and elongated, and the velum palati vibrated backward and forward during each respiratory act, as if it was wholly destitute of muscular control. The condition of the little sufferer was pitiable and extreme. With the assistance of Dr. W. S. Armstrong, I removed a portion of the uvula, hoping thereby to relieve the obstruction to free inspiration, and ordered a nauseant to be frequently administered, with a view of diminishing any spasmodic action of the larynx that might be present. This treatment failed to mitigate the severity of the symptoms in the slightest degree. I then commenced the treatment by electricity, using alternately the constant and interrupted galvanic and faradic currents, placing one pole at the back of the neck and passing the other—a steel-pointed electrode—along the palatine arches. The first application was marked by improvement—he slept well during the night. This treatment was continued at first daily, but as amendment progressed the intervals between the applications grew longer and longer, until he was dismissed, cured, in September last.

I could report other cases exhibiting results no less conclusive, but shall not trespass further upon your time or your patience; and I submit this record, as candid and as truthful as I have been able to make it, for your consideration. In a few, a very few, instances, I have been disappointed, but they were all cases of long standing; in some probably serious organic changes already existed, and most of them, having gone through various courses of treatment, resorted to this means as a forlorn hope. The salutary influence of electricity is by no means restricted to the brief catalogue of diseases enumerated in this paper, but its therapeutical application yields a rich harvest of valuable results in a much broader domain, and surgery, gynæcology, and obstetrics contribute their quota to the sum total of its grand possibilities, a bare mention of which would be incompatible with the limits of this article.

Now, gentlemen of the Academy, I ask is it wise, is it humane to disregard a remedy that has so much to commend it? Does our practice accord with our boasted liberality, which claims to exclude from our armamentarium nothing that can lighten the burdens or lessen the woes of suffering humanity? It is not offered either as a panacea or a specific; this is not claimed for it even by its most ardent advocates; yet I do not hesitate to affirm that its therapeutic results are as valuable, as certain, and as palpable as those of any remedy in the whole range of the materia medica.

